# Market-based control mechanisms for patient safety

**DOI:** 10.1136/qshc.2007.025833

**Published:** 2009-03-27

**Authors:** E Coiera, J Braithwaite

**Affiliations:** 1 Centre for Health Informatics, Institute of Health Innovation, University of New South Wales, Australia; 2 Centre for Clinical Governance Research, Institute of Health Innovation, University of New South Wales, Australia

## Abstract

A new model is proposed for enhancing patient safety using *market-based control* (MBC), inspired by successful approaches to environmental governance. *Emissions trading*, enshrined in the Kyoto protocol, set a carbon price and created a carbon market—is it possible to set a *patient safety price* and let the marketplace find ways of reducing clinically adverse events? To “cap and trade,” a regulator would need to establish system-wide and organisation-specific *targets*, based on the cost of adverse events, create a *safety market* for trading *safety credits* and then police the market. Organisations are given a clear policy signal to reduce adverse event rates, are told by how much, but are free to find mechanisms best suited to their local needs. The market would inevitably generate novel ways of creating safety credits, and accountability becomes hard to evade when adverse events are explicitly measured and accounted for in an organisation’s bottom line.

In 1999, the US Institute of Medicine published the landmark report “To Err is Human,” which outlined a strategy to reduce preventable medical error by 50% in 5 years.^1^ More years have passed, and we have yet to meet that target.^2^ One major unanswered question is how pressure and encouragement are to be applied to improve patient safety. Key contenders are litigation,^3^ safety training,^4^ systems change,^5^ legislation,^6^ information technology^7^ and accreditation.^8^ Pay-for performance (P4P), the linking of reimbursement with adherence to safety and quality measures, has received much recent attention and can produce modest to good improvements in quality,^9^ ^10^ but there remain difficulties:

- P4P targets a few behaviours or outcomes for reward, and can distract from non-targeted outcomes, focus on what is easily reportable or measurable^11^ and create uncertainty when one set of targets is replaced by another.^12^
- Clinical practice is highly localised, shaped by differing needs, practices, resources and cultural norms, and the variability of individual patients. Selecting meaningful, broadly applicable process or outcome targets is thus difficult. Further, targeting specific behaviours is a top-down strategy that does not adapt to local needs.
- There is no avenue for third parties to take a stake in quality improvement, hindering innovation and the harnessing of additional resources and skills unavailable in the clinical setting.

## A PROPOSAL FOR SAFETY UNDERPINNED BY MARKET-BASED CONTROL

Given our slow progress in improving the quality and safety of care, and the limitations of P4P, we propose a potentially radical new model for governing the safety of health services, inspired by successful environmental approaches. The US Federal Clean Air Acts of 1970 and 1990 set pollution-reduction targets but did not mandate how they were met. A central innovation was *emissions trading*, which allowed states to meet targets in highly flexible ways.

Emissions trading is also central to the Kyoto protocol, which has set a carbon price and created a market where carbon credits are traded between those who have reduced emissions below targets, and those who have not. While Kyoto has had little time to demonstrate its effectiveness, the US Clean Air Acts succeeded in dealing with acid rain, and the challenge of chlorofluorocarbons. There thus seems to be merit in using market-based mechanisms for other public good goals.

There are clearly similarities and differences between environmental and health regulation. Notably, both healthcare and the environment are public goods and involve the private and public sectors as participants. In contrast, a system like Kyoto is designed to engage nation states in a global regulation task, whereas health services could conceivably be regulated at a national, state or regional basis. Indeed, many of the most pressing challenges of the Kyoto protocol seem to have arisen for geopolitical reasons, including the inability of Kyoto nations to compel other nations to join the system, and the trade-offs that have to be accepted to get nations to sign, for example the divisions between how developed and developing nations are treated in the Protocol.

Emissions trading is however not a one-off governance model but rather is an example of market-based control (MBC).^13^ MBC systems were inspired by economic marketplaces but are now understood to be a general approach to optimising system responses to varying circumstances. MBC is thus a specific mechanism for system governance, not to be confused with ideological stances like “the free market.” MBC has been used widely and with much success, for example for the allocation of resources in communication networks. More generally, markets are used in many unexpected ways, such as the prediction of avian flu outbreaks and vaccine effectiveness. Defining attributes of MBC include decentralisation (there is no need for anyone to understand all the parameters being optimised) and distributed allocation of resources (individual agents make local decisions about allocation). Few measures are needed globally beyond the main controlling signal, be that price or quantity. Consequently, while we can draw inspiration from individual implementations of MBC, such as the Kyoto mechanism, it is probably more instructive to look at the more general attributes of MBC, and explore the types of MBC models that best suit the needs of healthcare, based upon first principles analysis and experimentation.

## DESIGNING A PATIENT SAFETY MARKET—“CAP AND TRADE”

So, can MBC be used to reduce avoidable patient harm? This has never been tried before. A safety-trading market might look something like this:

1. Set a *patient safety price*. A value is set on each preventable adverse event (PAE), based upon its estimated cost to the system.
2. Establish *system-wide targets*. Using an estimate of the current baseline PAE rate, set a global target and progressively reduce this over time.
3. Allocate *organisational targets*. For clinical organisations, estimate their current baseline PAE rate, and provide them with “credits” equal to a reduced target. Organisations must find ways to meet that target.
4. Create a *safety market* for trading *safety credits*. Organisations that reduce PAE rates below set targets have surplus credits, which they can sell. Organisations that miss targets must purchase market credits to meet target, or pay the regulator for credits at a set price. Failure to comply generates a large penalty.
5. Police the market through an *auditing mechanism*. Audit need not be universal but will need to be visible, credible, perhaps random to ensure compliance and responsive to attempts to distort, cheat or evade.
6. Allow for *certification* and audit of authorised third-party organisations. New organisations can independently initiate verifiable harm reduction projects to generate new credits.

**Figure 1:**
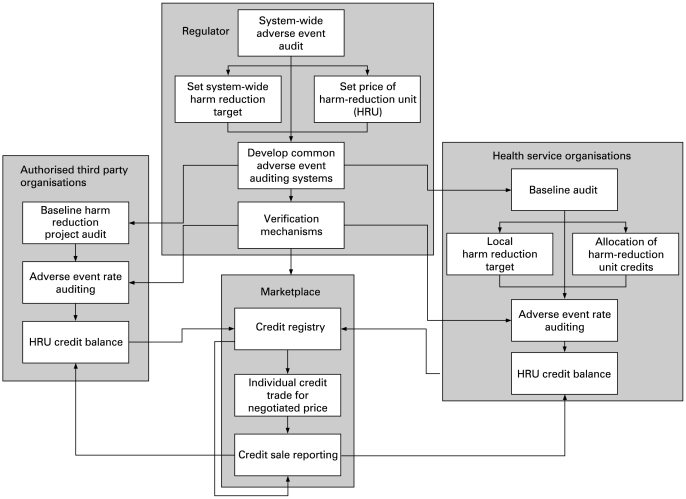
Governing a patient safety market: how the major players might align.

Figure 1 shows a possible safety market structure. The players include a regulator, who polices market mechanisms, health service organisations that meet harm reduction targets and authorised third-party organisations. The marketplace allows for the verifiable exchange of credits.

## MEASURING AND PUTTING A PRICE ON SAFETY

Central to the safety market are pricing and measurement mechanisms. The Agency for Healthcare Research and Quality (AHRQ) has defined 20 hospital and seven area-level safety indicators,^14^ and P4P programmes lead the way in identifying meaningful safety process and outcome measures, although this is an ongoing research area.

MBC would require such diverse safety indicators to collapse into a few, and perhaps just a single control variable. Kyoto targets, for example, are expressed in a single unit, a tonne of CO_2_, which is associated with a “carbon price” but actually covers a basket of *six* separate greenhouse gasses, accounting for 92% of global warming.^15^ Other gases are excluded because of concerns about their measurement. A weighting index converts the impact of each gas into a CO_2_ equivalent, so that they are interchangeable on the market. The resultant tradable commodity is sometimes known as an emission reduction unit (ERU).

**Figure 2:**
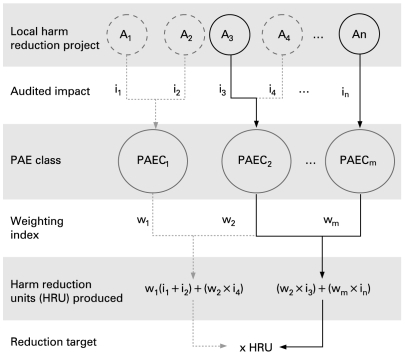
Calculating safety credits. Two separate clinical organisations institute different harm-reduction projects, aimed at reducing locally relevant causes of regulated preventable adverse event (PAE) classes. Project impacts in reducing the incidence of PAEs are audited, and then a weighting index converts PAE reductions into a common costable metric, such as days in length of stay prevented. The aggregate cost of all projects for an organisation, expressed as the total number of harm reduction units (HRUs) achieved, is then compared with the organisation’s target HRUs. Surplus HRUs generate tradable credits, to be bought by organisations that miss targets.

Can we develop a similar tradable patient *harm reduction unit* (HRU) (fig 2)? Could we identify a “basket” of leading PAE classes to cover a good proportion of clinical practices, and associate each class with a common measure of resource consumption, which can be assigned a cost? Recent work suggests that such an approach is indeed possible. We now know that a wide variety of PAE causes can be collapsed into a smaller basket of “natural” outcome categories, each category associated with a cost (additional days length of stay).^16^ Runciman *et al*’s analysis of 15 000 hospital admissions also found that 25% of PAE resources were expended on the 11 most frequent PAE categories (table 1), 50% by the top 45. Any such basket of PAEs is bound to be revised as we better understand how it relates to safety outcomes.

**Table 1:** Top “natural” categories of adverse event in hospital, and costs in terms of additional length of stay (from Runciman *et al*^16^)

Top principal adverse event category	Mean additional length of stay (days)	No of events in each category	Total no of extra days in hospital
Ongoing pain/restricted movement following back surgery	22	22	474
No, delay, inadequate investigations ischaemic heart disease	13	34	451
Wound infection following peripheral procedure	11	29	314
Incisional hernia: postprocedural	10	27	271
Postoperative bowel obstruction/adhesions	13	21	271
Injury due to fall in nursing home	12	19	219
Failed/blocked/ruptured/aneurysm, vascular grafts	13	17	215
Recurrent incisional hernia	9	20	190
Pulmonary embolism postoperatively	8	22	185
Wound infection following abdominal/retroperitoneal/pelvic procedure	5	35	178
Catheter-related urinary-tract infection	5	37	174

Each of these outcome categories can arise from a wide variety of different causes.

There are several benefits of a composite indicator like the HRU over simpler more reductionist measures such as the Hospital Standardized Mortality Rate (HSMR). First, an HRU based, say, upon length of stay is a much more fine-grained measure of clinical practice. Many of the adverse events in table 1, for example, would not translate into significant changes in a service’s mortality rates. Second, the composite nature of the HRU allows it to be more specifically targeted to the practices of different sectors, via the basket of PAEs chosen for inclusion. For example, the PAE basket for primary care is likely to be quite different to that used to monitor care in the acute hospital sector. Such differences may also come some way to explaining why a single measure like the HSMR is neither a consistent nor a reliable measure of quality.^17^

## ESTABLISHING A SAFETY BASELINE AND SETTING TARGETS

Both P4P programmes and carbon markets have explored different approaches to establishing performance baselines and targets. *Absolute targets* set identical goals across all organisations—for example, each hospital must reduce HRUs by 5000 each year. *Relative (intensity) targets* are expressed as a rate relative to a variable that models the intensity of clinical activity (eg, number of patients), and provide a more flexible mechanism, normalised to different scale organisations or patient loads. Relative targets can be set using multiple variables. For example, casemix modelling of individual organisations could adjust for the relative complexity of patient conditions or services provided.

There are risks and benefits to the different approaches that need to be considered when selecting baseline and target mechanisms. Experience with Kyoto suggests that fast-growing nations prefer relative targets, shrinking ones absolute targets.^18^ A related phenomenon is “grandfathering” or zero-price allocation of credits to existing organisations based upon their current performance. An organisation with poor safety performance could be relatively advantaged over an organisation with lower PAE rates, if past reductions at the safer organisation are not taken into account. Relative targets may mitigate against this but have their own limitations. As organisations grow, baseline PAE estimates also increase, generating new credits for expected additional PAEs. If the goal is to reduce the absolute number of PAEs, then intensity targets may be problematic. A number of studies show that, rather than issuing credits, auctioning them and recycling of revenue through tax reduction may lead to economic gains.

*Concentration* targets can define the period over which absolute or relative targets are met, and *contraction and convergence* targets allow organisations with widely different starting-points time to converge, first on absolute targets, and then switch to relative targets. The length of commitment to targets before baselines are re-estimated is also an issue. Experience with climate change suggests that 5 years best fits the life-cycle of policymakers.

Once trading is enabled, local auditing is needed to measure whether targets are met. The responsibility for developing and instituting such monitoring, reporting and verification systems will require thought.

## BENEFITS, RISKS AND POSSIBILITIES

We anticipate many benefits from a patient safety market. Organisations are given a clear policy signal to reduce PAE rates, are told by how much but are free to use whatever mechanisms best suit their local needs. The reward for excellent organisations that beat targets is that spare credits can be sold, yielding a financial reward that can be reinvested in improving care.

However, are we allowing unsafe organisations to buy their way out of trouble? In reality, an organisation that buys credits is not rewarded for poor performance but pays a transparent financial penalty. It would be difficult to sustain an organisation that has to spend money to prop up poor practice. The incentive to change is direct and unavoidable, and accountability hard to evade when PAEs are measured in the bottom line.

There is also significant opportunity for new players to enter the market. Businesses may emerge to exploit ways of minimising PAEs. A manufacturer of computer prescribing systems could audit their impact on medication errors, and the number of safety credits generated if a system were installed. Remotely monitoring the health status of older people at home could demonstrate a reduction in falls due to early intervention generating safety credits. Entrepreneurs could pay consumers to wear medication allergy armbands by demonstrating the bands reduce PAEs due to inappropriate medication. *Brokers* could help organisations find credits, agree a price, arrange their purchase and meet regulatory requirements. *Aggregators* could help smaller organisations with similar safety profiles to work together, sharing experiences, minimising resource utilisation on harm reduction projects and maximising their ability to generate credits.

To develop and test this new approach, we must understand the impact of the different possible market settings and ensure that benefits of error reduction strategies outweigh costs. We need to recognise that there is not a single market design but that a broad spectrum of design and implementation options are available, ranging from lighter weight models through to large, expensive and potentially heavily bureaucratic implementations. The evidence for the most effective market settings and designs will come from a number of sources. First, we need to learn from existing MBC approaches such as carbon trading. Careful consideration needs to be given to current experience with baseline-setting mechanisms, the regulator’s role, auditing and verification procedures so that consensus can be reached on these issues by the healthcare community, as well as on the structure of the marketplace itself (will it be a registry, a clearing house or a trading floor?). The market should ensure that large organisations do not distort credit prices. Some strategies will require early retirement of capital stock and generate short-term losses, or acquiring expensive new systems like electronic health records, while benefits are longer term.

Exploring the costs and benefits of different points in this large space of policy and implementation options is unlikely to be feasible through large-scale trials alone. In silico computer simulations offer a powerful alternate experimental model, which can help test out these many different market variations quickly and cheaply.^19^ Once there is sufficient evidence from simulations and other analyses that a particular MBC model shows promise in principle, the next step will undoubtedly require one or more small pilot studies. Pilot studies would be followed by larger-scale trials, for example using cluster randomisation, at a regional level among hospitals, to ensure that we are making evidence-based policy about harm reduction.

## OVERCOMING OBJECTIONS

We anticipate objections. Should we leave something as important as safety to a market, when governments have so clear a responsibility? Is it right to treat safety as a tradable commodity? One response is that health systems have tried many other strategies, but the patient safety problem seems entrenched and policy-resistant.^20^ The point is not whether commoditisation of safety leaves us feeling uncomfortable but whether it works. Others may point to criticisms of Kyoto that suggest better abatement can be achieved through taxes, or new technologies, and that too is a debate worth having.

Another major source of objection is the potential for MBC to introduce complex measurement, verification and trading systems, with significant compliance costs and with the risk of building a bureaucracy that is expensive, and could become disconnected from, and in conflict with, the clinical services it is meant to be helping. With much evidence now pointing to the need for clinicians to be actively engaged in quality improvement for it to be successful, the design of any MBC administrative structure needs to ensure it is well suited to the culture and nature of clinical services. However, it is also worth reminding ourselves of the current state of affairs. It has been estimated in the USA that adults receive recommended care just over half the time (55%)^21^ and for children, just under half the time (46%).^22^ Composite indicators for safety and quality that track improvement over time show that the rate of response to current safety and quality programmes is unacceptably slow.^23^ Clearly, poor quality and unsafe care remains a profound cost to the health system. As long as the benefits significantly outweigh costs, we believe that the size of any MBC system should not of itself be an issue.

So, in short, market forces, aphoristically labelled “the invisible hand” by Adam Smith, can be extremely efficient and powerful drivers of change. MBC may be the key to effective healthcare reform, promoting safety and error reduction, and may become a widely used governance mechanism, directed at many aspects of healthcare service delivery.
